# Baseline evaluation of nursing students’ informatics competency for digital health practice: A descriptive exploratory study

**DOI:** 10.1177/20552076231179051

**Published:** 2023-05-30

**Authors:** Kalpana Raghunathan, Lisa McKenna, Monica Peddle

**Affiliations:** 1 School of Nursing and Midwifery, 2080 La Trobe University, Bundoora, Victoria, Australia; 2 School of Nursing and Midwifery, 2104 Deakin University, Burwood, Victoria, Australia

**Keywords:** curriculum, digital technology, education, informatics competency, nursing, students, self-assessment

## Abstract

**Introduction:**

The healthcare system is increasingly technology-dependent and proficiency in informatics skills is essential for health professionals to efficiently operate in the contemporary clinical environment. Nurses are major users of digital health technologies and graduates need to be well-prepared and confident to use the different available clinical systems competently as they transition from education to practice.

**Aim:**

To explore undergraduate nursing students’ self-perceptions of informatics competence, set within a larger research project.

**Method:**

Descriptive, exploratory cross-sectional research design, with online self-assessment survey of undergraduate nursing students (*n*  =  142). Data were analysed with descriptive, correlation and comparative statistics.

**Results:**

Participants’ perceived overall mean informatics competency was at the level of somewhat competent, with only 40.84% (*n*  =  58) at the level of competent. The highest mean value was in foundational information and communication skills and the lowest in information and knowledge management. Formal informatics education within curriculum was limited and lacked uniformity, as was prior exposure to important simulated informatics tools in preparation for practice. Factors including academic year level, computer experience and previous experience using clinical systems had a significant impact on participants’ perceived informatics competency.

**Conclusion:**

Even though informatics competence is vital for clinical practice, with technology becoming pervasive within healthcare, nursing students’ preparedness for digital health was sub-optimal. There were gaps in students’ critical informatics practice knowledge with implications for work readiness of future graduates and nurse education practice.

## Introduction

Increasingly, information and communication technologies (ICTs) such as electronic medical records (EMR) and clinical information systems have been adopted across the healthcare system to increase efficiencies, improve workflows and promote safety and quality in care delivery.^1^ These digital health technologies are an integral part of the healthcare environment associated with considerable benefits including the storage and management of vast amounts of health data, coordinated patient care, improved access and quality of patient data and improved interdisciplinary health team communication.^2,3^ Given that nurses are at the frontline of care and major digital technology users, informatics is a core professional competency for clinical practice.^4^ Nurses need to be knowledgeable and skilled to competently use the different health informatics tools to assist care.^5,6^ Broadly, informatics competencies include abilities to use ICT, data and information management skills effectively and appropriately in the technology-enabled work environment to improve patient care and health outcomes.^7^

In response to the growing digitalisation of healthcare, the need for informatics competencies in nurse education to prepare future graduates for the technology-enabled work environment is internationally recognised.^8,9^ Moreover, given the expansion of digital tools healthcare services expect technology-ready graduates entering the professional environment.^10,11^ It is, therefore, important that students are confident and comfortable in using digital technologies safely and effectively to access and use evidence-based data to support nursing care in the clinical environment.^5^

### Background

With growing emphasis on ICT in health, nursing students’ informatics and digital proficiencies in entry-to-practice have become a major focus within academic preparation in recent years.^12,13^ Progressively, curriculum reforms to integrate informatics content and increased investment into learning technologies, such as simulated EMRs, have been associated with educational interventions to improve students’ readiness for digital practice.^14,15^ However, significant gaps in nursing graduates’ readiness for technologies in the clinical setting have been reported internationally.^16,18^ A recent Australian study of final-year nursing students’ preparedness for EMRs in clinical practice showed that a majority (71.1%, *n*  =  70) did not feel confident to use EMRs in the healthcare setting.^19^ Another study about digital literacy among nursing students in an Australian university showed that only 55% (*n*  =  84) considered their overall applied computing skills as competent for clinical settings.^20^ In addition, although health professions students have reported high levels of basic, everyday digital literacy, this competence did not translate into the clinical practice context.^21,22^ Furthermore, despite being digitally savvy, and students generally accepting digital technologies positively, there are reported deficits in confidence to use clinical systems.^23^

Previous examinations of nursing students’ perceived informatics knowledge showed that they were predominantly at a basic level, and students were most confident with basic ICT skills.^24,25^ Students did not perceive themselves as competent in content areas such as applied computer skills and clinical informatics role.^24^ In addition, despite curriculum preparation, students lacked competence in vital knowledge, not being confident around professional expectations including the application of informatics concepts into practice.^26,27^ Alarmingly, even the most recent evaluation of final-year nursing students in a Canadian study showed overall perceived informatics competency and confidence in their abilities to use digital tools remained at the level of somewhat competent/confident (mean [SD] 2.93, 0.46).^28^ Kleib et al. identified gaps and inconsistencies in students’ understanding of informatics and digital health practice even with informatics education. Like earlier reports, Kleib et al. found this cohort was most confident in foundational ICT skills (mean [SD] 3.27, 0.50); reporting less than competent mean scores in information and knowledge management (mean [SD] 2.79, 0.52), professional responsibility/accountability (mean [SD] 2.84, 0.57) and use of ICT in delivery of care (mean [SD] 2.97; 0.59) domains.

Given the importance of informatics capabilities for contemporary practice and an evolving healthcare environment, ongoing cross-sectional self-assessments of competence are important to assess nursing students’ work-readiness as they prepare to transition from education to practice. Furthermore, establishing a baseline measure of competence among nursing students is essential to determining curriculum-wide improvements for optimal preparation within academic programs.^6,25^ The aim of this study was to explore undergraduate nursing students’ self-perceptions of informatics competence. It is a sub-study of a doctoral multi-methods research project investing in curriculum preparation for digital health in pre-registration nurse education in Australia. Other studies from this larger investigation are reported elsewhere. The research question framing this current inquiry was: How do pre-registration nursing students perceive their preparation for using digital technologies to deliver health and care in the clinical environment?

## Methods

### Study design and ethics approval

A descriptive, cross-sectional study was used to determine perceived informatics competency and readiness for digital health practice among pre-registration nursing students through an online self-assessment survey. Ethics approval for the study was obtained from La Trobe University Human Research Ethics Committee (HEC21254).

### Survey instrument

Survey instrument selection for the study was facilitated by a rapid review of nurse education literature and critical evaluation of reported validity confirmed self-administered instruments to measure the informatics competence of nursing students.^29^ Based on its strengths, the Canadian Nursing Informatics Competency Assessment Scale (C-NICAS) was selected and adapted with permission from the authors.^30^ This 21-item scale demonstrated sufficient evidence of validity and reliability, applicability across different settings and targeted entry-to-practice level competencies. Its authors reported high internal consistency reliability for the C-NICAS and its four subscales; overall Cronbach's alpha coefficient for the scale was 0.93; foundation ICT skills (three items), 0.81; information and knowledge management (six items), 0.85; professional and regulatory accountability (six items), 0.81; and use of ICT in delivery of care (six items), 0.87. Instrument features including standardisation, scale length, and its competency evaluation indicators aligned with informatics and digital health capability expectations for nurses in Australia^31,32^ supporting its suitability to use in this study.

In Part 1 of the survey, 23 general non-identifiable contextualised demographic questions collected information about sample characteristics and current informatics and ICT experience. Part 2 contained the original 21 C-NICAS self-assessment questions scored using a 4-point Likert scale rating from not-competent to competent (1  =  not competent; 2  =  somewhat competent; 3  =  competent; 4  =  very competent) for each competency (see Supplementary materials). For this study, a minor addition involved contextualisation by expanding some examples supporting competency indicators to localise terminology and enhance comprehension. Instrument content validity was assessed through face validity for comprehensiveness, comprehension and suitability in this study with nursing faculty experts (*n*  =  2); pilot testing with nursing and midwifery school doctoral student volunteers (*n*  =  10); as well as statistician verification of accuracy and suitability of questions and measurements used to minimise measurement bias, namely instrument bias.^33^ Feedback was positive; therefore, no changes were made to the measurement criteria or the scale.

### Sample and recruitment

The target population in this study was pre-registration nursing students from Bachelor of Nursing (BN) programs preparing Registered Nurses (RN), usually conducted in universities, and second-tier Diploma of Nursing (DN) programs preparing Enrolled Nurses (ENs) (second-level nurses) from vocational education settings in Australia. Using non-probability convenience sampling, eight nursing schools (five universities; three vocational education institutions) were contacted to assist data collection in this study, and three schools located in Victoria agreed to participate through Dean or Head of School. Bachelor of Nursing (BN) students from years 1 to 4 in single- and double-degrees at two universities and Diploma of Nursing (DN) students from one TAFE (Technical and Further Education) institution were invited to participate. Both universities had main campuses in metropolitan and regional locations. The DN program was delivered at a metropolitan campus. Based on a 95% confidence level, 0.5 standard deviation and margin of error (confidence interval) of ±5%, a sample size of 360 was determined as adequate for this study. Sample selection bias was minimised by including the entire population for potential inclusion. The study was advertised via the nursing schools’ online education platforms through appointed school contacts. Survey advertisement included an invitation letter and the URL link to the survey, which when clicked on, displayed an information page and an embedded consent statement. To obtain an acceptable response rate, three reminders were sent highlighting the importance of students’ input.

### Data collection and analysis

The survey was administered via REDCap software (Vanderbilt University, Nashville, Tennessee) to collect quantitative data between between September 2021 and April 2022. Participation was voluntary with no incentives provided. Completing the survey online indicated consent; it was anonymous and required approximately 10 min to complete. Participants could exit the survey at any time prior to clicking on the submit button. Once responses were submitted, individual URL survey links expired. Responses to the questionnaire could not be withdrawn once the submit button was clicked because it was not linked to any personal identifiers.

A data analysis plan and codebook were developed to assist statistical data analysis using IBM SPSS Statistics Version 28. Data cleaning was undertaken in Microsoft Excel file. Missing data was imputed by deriving mean value for continuous variables and frequency value for categorical variables and a value of zero for questions skipped when they did not apply to the respondent^34^; therefore, all cases were included in statistical analysis. Statistical techniques included descriptive analysis to report sample characteristics and scale results; correlation analysis and mean comparison t-tests and one-way ANOVA; internal consistency reliability and factor analysis for scale properties.

## Results

### Participant characteristics

A total of 143 participants responded, with 142 surveys retrieved for analysis, a 39.4% response rate which was below the forecasted sample size. A single DN survey was excluded because of curriculum difference and inability to meaningful analysis of the data about this cohort from a single survey. However, it was decided to include a single survey from a participating university because of the similarity in student cohort and nursing curriculum in that university. All included surveys were from BN programs.

Table 1 illustrates participant characteristics. Most participants (73.24%, *n*  =  104) were from a metropolitan campus; 71.83% (*n*  =  102) were enrolled in blended programs involving both face-to-face and online learning. The majority (98.59%, *n*  =  140) were enrolled in a three-year degree; 40.85% (*n*  =  58) were second-year students. Most participants (49.30%, *n*  =  70) were in the 20–25 years age group; 88.03% (*n*  =  125) were female which is consistent with the nursing student population in the BN program. About one-third (32.39%, *n*  =  46) completed school before enrolling in the nursing degree; 23.02% (*n*  =  33) were ENs completing the BN program. For a majority (84.51%, *n*  =  120), informatics was not taught formally in the curriculum. Participants with formal informatics instruction selected the following: health informatics course (0.70%), nursing fundamentals course (5.63%), multiple courses in the curriculum (0.70%), integrated across the curriculum (2.82%) and via a seminar or lecture (4.93%). Less than half (47.89%, *n*  =  68) had used EMRs previously, which included: simulated version EMR (3.53%), as part of current work (23.94%) or during clinical placement (38.03%). During clinical placements participants had used EMRs with individual student passwords (22.54%), alongside supervising RNs or facility staff (8.45%) or with clinical educators (3.52%). More than half (57.75%, *n*  =  82) self-rated their computer skill at medium level of proficiency; 69% (*n*  =  98) used computers several times per day. Furthermore, more than half (59.15%, *n*  =  84) had accessed or used their personal national electronic health record, known as My Health Record.^1^ A majority (73.94%, *n*  =  105) did not participate in online video gaming, and social media use varied. Facebook (30.03%), Instagram (40.08%) and TikTok (27.05%) were most frequently used, while Twitter and LinkedIn were either rarely or never used.

**Table 1.** Participant characteristics.

		N %	
Campus location			
	Metropolitan	104	(73.24)
	Rural regional	38	(26.76)
Study mode			
	On campus	25	(17.61)
	Online	15	(10.56)
	Blended	102	(71.83)
Year level			
	1	42	(29.58)
	2	58	(40.85)
	3	40	(28.17)
	4	2	(1.41)
Sex			
	Female	125	(88.03)
	Male	14	(9.86)
	Other	2	(1.41)
	Not disclosed	1	(0.70)
Age group			
	20–25	70	(49.30)
	26–35	38	(26.76)
	36–45	23	(16.20)
	46–45	9	(6.34)
	>55	2	(1.41)
Informatics education included in curriculum			
	Yes	22	(15.49)
	No	120	(84.51)
Previously has used EMRs			
	Yes	68	(47.89)
	No	74	(52.11)
Perceived computer skill level			
	Beginner	9	(6.34)
	Medium	82	(57.75)
	Advanced	51	(35.92)
Nursing program entry pathway			
	School leaver	46	(32.39)
	Graduate entry	36	(25.35)
	Enrolled Nurse	31	(21.83)
	Other	29	(20.42)

EMR: electronic medical records.

### Perceptions of informatics competencies

The range of informatics competency score was 1–4 with 3 being competent. Participants’ overall perceived informatics competency mean score was 2.77 (SD 0.67); 15.5% (*n*  =  22) self-rated as not competent, 41.45% (*n*  =  59); somewhat competent, 40.84% (*n*  =  58); competent and 2.11% (*n*  =  3) as very competent (Table 2). The highest mean value was in foundational ICT skills (mean [SD], 3.28 [0.62]); followed by professional and regulatory accountability (mean [SD], 2.83 [0.83]). The use of ICT in delivery of patient care was slightly lower (mean [SD], 2.60), [0.92]); information and knowledge management was lowest (mean [SD], 2.37 [0.85]).

**Table 2.** Nursing students’ overall perceptions for informatics competency.

	Scale: NI competency	Subscale: Foundational ICT skills	Subscale: Information and Knowledge Management	Subscale: Professional and Regulatory Accountability	Subscale: Use of ICT in Delivery of Patient Care
*N*	142	142	142	142	142
Mean	2.77	3.28	2.37	2.83	2.60
Median	2.88	3.33	2.33	3.00	2.67
SD	0.668	0.617	0.854	0.829	0.918
Minimum	1.17	1.33	1.00	1.00	1.00
Maximum	4.00	4.00	4.00	4.00	4.00
Not competent (%)	15.49%	2.11%	28.87%	13.38%	22.53%
Somewhat competent (%)	41.54%	18.30%	40.84%	29.57%	34.50%
Competent (%)	40.84%	58.45%	23.23%	45.07%	32.39%
Very competent (%)	2.11%	21.12%	7.04%	11.97%	10.56%

NI: nursing informatics; ICT: information and communication technology; SD: standard deviation.

#### Foundational ICT skills

**Figure 1.**
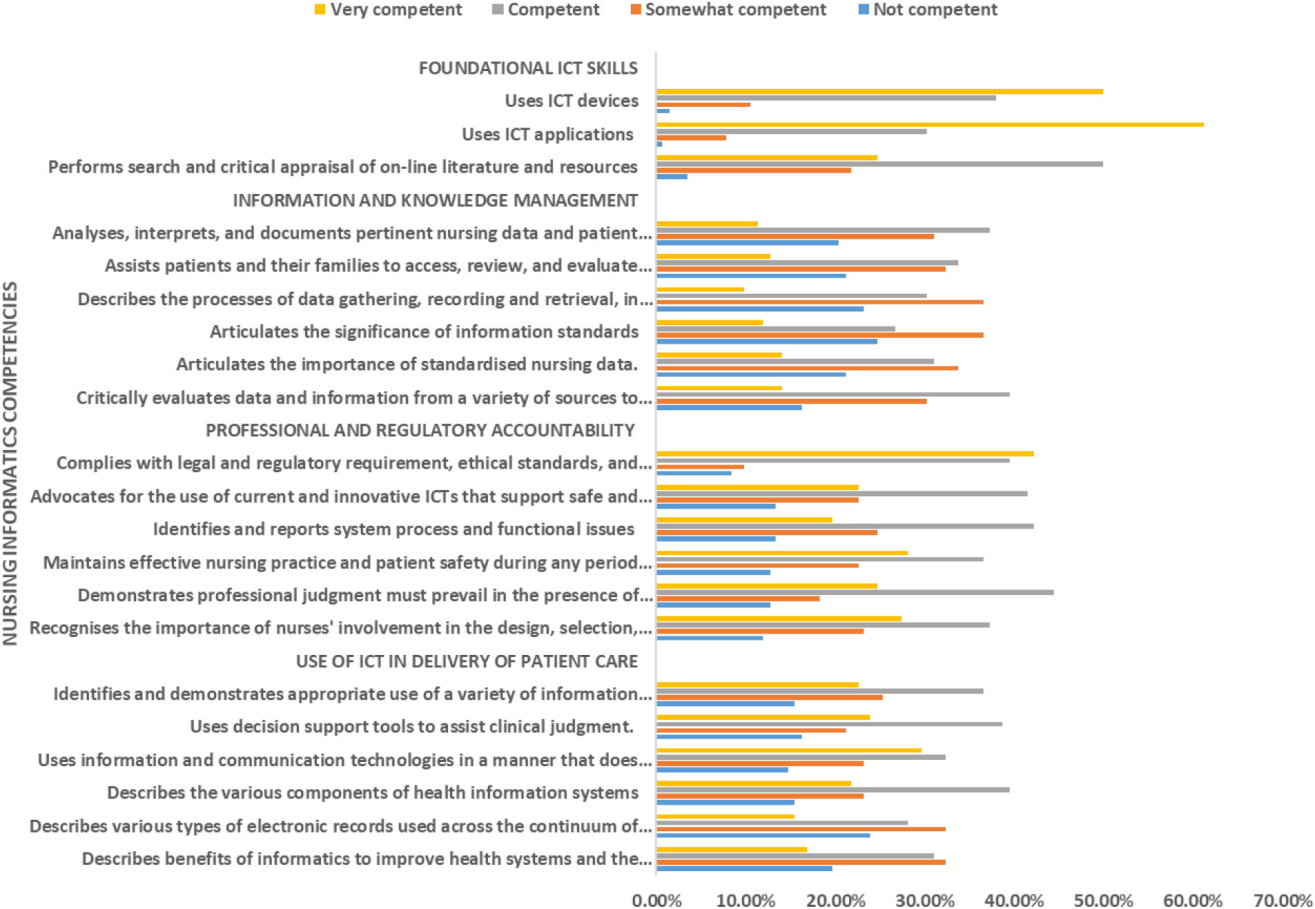
Nursing students’ self-evaluation of informatics competencies.

Table 3 illustrates mean scores for the C-NICAS scale items. Within foundational ICT skills participants rated ability to search and critically appraise online resources (mean [SD], 2.96 [0.78]) lowest; only half (*n*  =  71) reported being competent in this aspect. Mean values for the use of ICT devices (mean [SD], 3.37 [0.73]) and ICT applications (mean [SD], 3.52 [0.67]) were higher; 50% (*n*  =  71) indicated they were very competent with ICT devices; a majority (61.27%, *n*  =  87) was very competent with using ICT applications. Figure 1 illustrates nursing students’ self-evaluation of individual competencies.

**Table 3.** Central values for nursing informatics competencies.

Foundational ICT skills		*N*	Mean	Median	SD
1	Uses ICT devices	142	3.37	3.5	0.72
2	Uses ICT applications	142	3.52	4	0.67
3	Performs search and critical appraisal of on-line literature and resources	142	2.96	3	0.78
Information and Knowledge Management					
4	Analyses, interprets and documents pertinent nursing data and patient data using standardised nursing and other clinical terminologies	142	2.39	2	0.93
5	Assists patients and their families to access, review and evaluate information	142	2.38	2	0.95
6	Describes the processes of data gathering, recording and retrieval, in (electronic or paper records) and identifies informational risks, gaps and inconsistences across the healthcare system	142	2.27	2	0.93
7	Articulates the significance of information standards	142	2.26	2	0.96
8	Articulates the importance of standardised nursing data	142	2.38	2	0.97
9	Critically evaluates data and information from a variety of sources to inform the delivery of nursing care	142	2.51	3	0.92
Professional and Regulatory Accountability					
10	Complies with legal and regulatory requirement, ethical standards and organisational policies and procedures	142	3.15	3	0.91
11	Advocates for the use of current and innovative ICTs that support safe and quality care	142	2.73	3	0.96
12	Identifies and reports system process and functional issues	142	2.68	3	0.94
13	Maintains effective nursing practice and patient safety during any period of system unavailability	142	2.80	3	0.99
14	Demonstrates professional judgement must prevail in the presence of technologies designed to support clinical care	142	2.81	3	0.95
15	Recognises the importance of nurses’ involvement in the design, selection, implementation and evaluation of ICT applications and systems in health care	142	2.80	3	0.97
Use of ICT in Delivery of Patient Care					
16	Identifies and demonstrates appropriate use of a variety of information and communication technologies	142	2.66	3	0.99
17	Uses decision support tools to assist clinical judgement.	142	2.70	3	1.00
18	Uses ICT in a manner that does not interfere with the nurse-patient relationship.	142	2.77	3	1.03
19	Describes the various components of health information systems	142	2.68	3	0.98
20	Describes various types of electronic records used across the continuum of care and their clinical and administrative uses.	142	2.35	2	1.01
21	Describes benefits of informatics to improve health systems and the quality of Inter-professional patient care.	142	2.45	2	0.99

ICT: information and communication technology; SD: standard deviation.

#### Information and knowledge management

Under information and knowledge management, all six items displayed consistently low mean values. Participants rated item nine related to critical evaluation of data and information only slightly above other items in this domain (mean [SD], 2.51 [0.93]). Nearly a third (30.38%, *n*  =  43) indicated that they were only somewhat competent and 16.20% (*n*  =  23) not competent in this area. Lowest mean values were associated with item seven related to health technology information standards (mean [SD], 2.26 [0.97]), with more than a third (36.62%, *n*  =  52) being only somewhat competent, and one quarter (24.65%, *n*  =  35) not competent in relation to information standards.

#### Professional and regulatory accountability

All six items under professional and regulatory accountability showed higher means values than information and knowledge management skills. Item 10 pertaining to legal, ethical and organisational requirements displayed the highest score (mean [SD], 3.15 [0.92]; most participants selected very competent (42.25%, *n*  =  60) and a comparable percentage of 39.44% (*n*  =  46) selected competent. Participants rated their ability to identify and report system issues (mean [SD], 2.68 [0.94]) the lowest, but a majority (42.25%, *n*  =  60) indicated that they were competent, while 19.72% selected very competent (*n*  =  28).

#### Use of ICT in delivery of patient care

Six items under the use of ICT in delivery of patient care were also rated above information and knowledge management skills. Highest mean value was associated with item 17 related to the use of ICT and nurse–patient relationship (mean [SD], 2.77 [1.04]), with a large portion of participants indicating that they were competent (32.39%, *n*  =  46) and a comparable number selected very competent (29.58%, *n*  =  42). Mean value for item 20 pertaining to knowledge of various types of electronic records used in healthcare (mean [SD], 2.35 [1.01]) was the lowest; about a third (32.39%, *n*  =  47) indicated that they were only somewhat competent; 23.94% (*n*  =  34) selected not competent.

### Variables explaining perceived informatics competencies

For meaningful analysis against year level, data was collapsed by combining two cases from the double degree-fourth year into third year (final year) since the distribution of responses closely matched. Relationships between variables were investigated using Pearson product–moment correlation coefficients. Data showed no significant relationship between perceived informatics competencies and the variables: campus location, study mode, sex, age, form of prior informatics education, frequency of computer use, entry pathway and education level. Correlation was significant at the *p*  ≤  .05 level with moderate positive correlation for year level (*r*  =  .34, *p*  ≤  .001) and computer experience (*r*  =  .36, *p*  ≤  .001). There was moderate negative correlation with previous EMR experience (*r*  =  −.36, *p*  ≤  .001) and being an EN (*r*  =  −.17, *p*  =  .03). Multiple regression analysis showed that year level (*p*  =  .026), computer experience (*p*  ≤  .001) and prior EMR experience (*p*  =  .014) made a significant contribution to the prediction of perceived informatics competencies and explain a statistically significant 28.4% (*R*^2^  =  .284) of the variance in perceived informatics competencies. Being an EN (*p*  =  .15) did not make a significant contribution to the prediction of informatics competencies. Among these three predictor variables, computer experience made the larger contribution explaining 9% of variance, over year level (4%) and prior EMR experience (4%).

### Demographic characteristics and perceived informatics competencies

Independent-sample t-tests were used for two-group comparisons which showed a statistically significant difference was reached in means scores for previous EMR experience (*p*  ≤  .001) and EN characteristic (*p*  =  .03) (see Table 4). The extent of difference in means for previous EMR experience was moderate (Cohen's *d*  =  0.78) and did not significantly impact mean score for foundational ICT skills (*p*  =  .10; mean difference  =  .27). There was a statistically significant difference for information and knowledge management (mean difference  =  .40, *p*  =  .01), professional and regulatory accountability (mean difference  =  .47, *p*  ≤  .001) and use of ICT (mean difference  =  .78, *p*  ≤  .001). The extent of difference in means related to EN qualification was small (Cohen's *d*  =  0.42) and did not reach statistically significant difference for individual subscales; highest mean difference was in the use of ICT (mean difference  =  .47, *p*  =  .13).

**Table 4.** Independent-sample t-test comparing groups and informatics competencies.

Groups		*N*	Mean	SD	Mean difference	95% CI		*df*	*t*	*p*	Cohen's *d*
Previously used EMRs											
	Yes	68	3.02	0.49	0.48	0.28, 0.69		129	4.72	<.001*	0.78
	No	74	2.53	0.72							
Enrolled nurse											
	Yes	33	2.98	0.64	0.27	0.02, 0.53		54	2.11	0.03*	0.42
	No	109	2.7	0.66							

* The mean difference is significant at the 0.05 level (two sided).

M: mean; SD: standard deviation; CI: confidence interval; EMR: electronic medical records; EHR: electronic health records.

One-way analysis of variance for groups with three or more categories showed statistically significant difference in overall scores for year level (*p*  ≤  .001), computer experience (*p*  ≤  .001) and entry pathway into nursing (*p*  =  .03) (Table 5). The extent of difference in the year-level means was large (eta squared  =  .12). Post-hoc comparisons using Tukey HSD test indicated that the mean score for year 1 was significantly different from that of years 2 and 3; Year 2 did not differ significantly from year 3. For computer experience, the extent of difference in means was also large (eta squared  =  .14) and Tukey HSD test indicated significant differences in mean scores between each category (beginners, medium, advanced). For nursing program entry pathway, the extent of difference in the means was only moderate (eta squared  =  0.06) and Tukey HSD test indicated that the mean score for two categories was significantly different: enrolled nurse (mean [SD], 3.02 [0.61]) and other pathways (mean [SD], 2.51 [0.71]).

**Table 5.** One-way analysis of variance comparing participant characteristics and informatics competencies.

Categories		*N*	Mean	SD	95% CI		*F*	*p*	Eta squared
Year level									
	1	42	2.43	0.81	2.18, 2.68		9.7	<.001*	0.12
	2	58	2.82	0.56	2.67, 2.97				
	3	42	3.03	0.49	2.87, 3.18				
Computer skill level									
	Beginner	9	2.06	0.73	1.50, 2.61		11.39	<.001*	0.14
	Medium	82	2.68	0.63	2.54, 2.81				
	Advanced	51	3.03	0.60	2.86, 3.20				
Nursing entry pathway									
	School leaver	46	2.74	0.69	2.54, 2.95		3.04	0.03*	0.06
	Graduate entry	36	2.78	0.58	2.58, 2.98				
	Enrolled Nurse	31	3.02	0.61	2.80, 3.24				
	Other	29	2.51	0.71	2.24, 2.78				

* The mean difference is significant at the 0.05 level.

M: mean; SD: standard deviation; CI: confidence interval.

### Instrument psychometrics

Data was considered suitable for factor analysis based on five cases to each item ratio for small sample sizes,^34^ Kaiser–Meyer–Olkin measure of sampling adequacy (KMO  =  .932), the Bartlett's Test of Sphericity (*p*  =  .000) and a higher proportion of correlation coefficients of .3 and above in the Correlation Matrix. Using Principal Component Analysis (PCA) four factors were extracted. The first four components recorded eigenvalues above 1 (12.265, 1.917, 1.575, 1.110) and explained a total of 80.32% of the variance. Internal consistency reliability was high for the overall scale (Cronbach's alpha 0.96) and its four subscales (Cronbach's alpha 0.81, 0.95, 0.93, 0.96) recording Cronbach's alpha values above 0.7 considered acceptable.^34^ Scale inter-item correlation mean was .54 ranging from .13 to .87.

## Discussion

The results of this study show that nursing students’ overall perceived informatics competency in preparation for digital practice were at the level of ‘somewhat competent’ like the findings of Kleib et al.'s^28^ Canadian study. Students in the current study were found to be most confident in foundational ICT skills, with lowest mean score in the information and knowledge management domain. However, unlike Kleib et al., in our study, students self-rated their knowledge in professional and regulatory accountability as higher than the use of ICT in delivery of patient care. Our findings also reflect earlier reports wherein students were most confident in basic ICT skills and less confident in critical knowledge involving applied informatics skills.^24,27^ Results confirmed previous reports that students were less confident in the knowledge of clinical systems within the healthcare environment despite high levels of basic digital literacy.^19,20,22,23^ Even though the difference between the total percentage of students who were somewhat competent and competent was negligible, and that nursing students were in the process of developing informatics capabilities, overall results clearly indicate concerning gaps in their preparedness for digital health. Despite the passage of time since informatics education gained importance,^8,10,12^ and notwithstanding the ubiquitous use of digital technologies in contemporary healthcare, deficits in students’ core informatics practice knowledge and skills persist. It could be argued that while students showed general awareness of informatics, they were underprepared for the critical knowledge necessary to apply informatics in patient care or clinical context.^28^ This suggests current strategies towards informatics development in the undergraduate curriculum are insufficient requiring further education reform.

In the current study, it was also concerning that most participants reported not receiving informatics education in their curricula. Moreover, when instruction was provided, different approaches were evident exposing discrepancies in students’ preparation. These variable educational experiences can further compound problems with students’ inadequate informatics preparation.^35,36^ These findings have implications for nursing practice and education, highlighting significant curriculum lag and that current informatics preparation in undergraduate curricula lacked uniformity. Part of the problem could be that technology developments have been rapid and nurse education, and more specifically curriculum reforms, have not kept pace.^3^ In addition, any major curriculum revision is a complex process requiring significant resources including expertise among faculty to teach informatics which may be currently lacking.^35^ Furthermore, although the health workforce roadmap and nursing accreditation standards in Australia stipulate informatics development, the absence of national entry-to-practice informatics competency guidelines complicates efforts in standardisation of curricula to ensure consistent graduate preparation.^11^ Until such national initiatives are available to drive comprehensive curriculum reform, informatics integration is likely to remain fragmentary within undergraduate curricula. Nevertheless, the results of our study highlight opportunities for academic programs to consider strategies to integrate informatics instruction including greater exposure to major digital technologies in preparation for practice. Since it is the responsibility of academic institutions to prepare future graduates, nursing schools must proactively initiate curricula reforms to keep pace with technology-related changes in the healthcare system. But there is also the need for more guidance from education policy around the expected level of informatics preparation within nursing curricula. Having documented criteria for informatics education can mitigate inconsistencies in students’ academic preparation, as well as help academia to identify the necessary content within curriculum preparation.^8,11^

The current study revealed inconsistencies and disparities in students’ knowledge of informatics content areas. In relation to foundational ICT skills, it was unsurprising to find that a vast majority rated themselves as very competent based on what is already known about students’ digital literacy^22,23^ and that these skills are indispensable with the pervasive use of ICT in everyday life. But it was interesting to find that while a majority considered themselves as very competent with ICT applications and devices, only half felt confident in their ability to search and critically use online data sources which involved applied knowledge to access and use evidenced-based resources, skills that were central to health professional practice and foundational nursing skills^5,7^ taught in existing curricula. One strategy that could assist schools to address consistency with foundational skills is a baseline evaluation of digital literacy before student cohorts commence the course or at the start. Such an evaluation could be beneficial to initiate appropriate remedial interventions in the form of preparatory ICT skills.^23^

Regarding information and knowledge management, a core informatics competency,^7^ it was worrying that more than half were somewhat competent or not competent reflected in scores across this domain. Nurses have a major role in collecting, documenting and managing health data and information crucial for timely clinical decisions and multidisciplinary care delivery; and it is imperative that nursing students, not only know how to use digital technologies but also have a comprehensive understanding of skills needed to manage health data and information necessary to generate new knowledge and wisdom supporting informed clinical decisions.^7^ Therefore, rectification of this identified gap in students’ knowledge is urgent. In addition to curricula content revision, a practical solution to address this competency area is to increase students’ exposure to different ICT-based applications in the academic environment including the use of online learning tools, evidence-based reference resources, scholarly databases and supplementary digital learning resources which can help improve information management skills.^37,38^ Results across professional and regulatory accountability were encouraging, and a majority felt competent or very competent in this critical practice area. Professional and regulatory knowledge effecting practice is not only a crucial component of broader health professionals practice standards but they are also central to safety and quality care with critical impact on healthcare services and patients’ health outcomes.^3^

With the use of ICT in delivery of patient care although students’ mean score was only somewhat competent, a majority considered themselves competent in appropriate ICT use and the nurse–patient relationship, using decision support tools to inform clinical judgement, as well as knowledge about various aspects of health information systems. However, more than half felt that they were only either somewhat competent or not competent in knowledge around different EMR systems and the benefits of informatics for healthcare systems and inter-professional patient care. This result is significant given the widespread use of digital systems across healthcare and all-important emphasis on collaborative care from health team members.^3,14^ More significantly, as highlighted by Kleib et al.,^4^ this knowledge shortfall not only indicates gaps in nursing students’ technology readiness but it could also mean that the full benefits of implemented digital technologies may not be realised if as clinicians they lack comprehensive know-how to use them meaningfully and optimally in the clinical context. Therefore, it is vital that students are introduced to major point-of-care informatics tools such as EMRs in the academic setting. The use of simulated or academic EMRs within the nursing curricula can be valuable practical teaching and learning tools in preparation for technologies used in the clinical context.^28,39^

The current study revealed year level, computer experience and previous EMR experience had a significant impact on students’ perceived informatics competency. Prior EN qualification and entry pathway also had an effect, but not a significant one. There was a statistically significant correlation between computer experience, year level and previous EMR experience and total informatics competency score. The positive association between higher computer experience and higher informatics competency is not surprising since students are likely to be more confident in their ICT skills because of prior knowledge around computers, but again this does not necessarily translate into greater understanding of informatics concepts for practice.^27,40^ Brown et al.^20^ found that competence increased as student cohorts progressed academically, and competency also increased among students who had previously used EMRs during practicum or in paid employment. In the current study, less than half of the students had any prior EMR experience which could be the reason why students lacked sufficient knowledge about different EMR systems used in healthcare and actual utilisation between the health team. A large part of prior exposure was reported in clinical placements, with minimal preparatory simulated EMR experiences. This finding is supported by previous research showing underutilisation of simulated EMRs in nursing programs.^39^ There are problems with reliance on clinical placements for students to gain informatics-related skills for reasons including varied experiences and limited student access to clinical venue EMRs which contributed to students feeling underprepared for digital technologies.^19,36^ This issue again highlights the need for students to have exposure to important digital technologies within the learning environment. Such early training can significantly contribute to students’ overall informatics competencies and transition to the clinical setting.^18,19^ Moreover, given that it is an important for safety and quality care, students should have prior technology preparation before entering the clinical setting and introduced to the actual systems that they will use in their routine work practice later as graduates.

Our results showed that age, prior informatics education, and education level did not have a significant effect on informatics competency. This result contradicts that of Kleib et al.^41^ who noted a statistically significant difference in informatics competency among Canadian nurses in relation to all demographic characteristics, including education level, suggesting a likelihood for increasing competence with more education. Although it could be argued that given the population in the latter study were practicing nurses with different clinical experience and practice competencies, therefore, factors related to informatics competency could be different. But our result concurs with Zamarripa-Zoucha's^27^ US-based study of nursing students at different levels of university education. Zamarripa-Zoucha did not find statistical significance between students’ education level and informatics competency. However, the lack of association between previous informatics education and total competency score observed in our study contradicts Zamarripa-Zoucha's positive association between participation in informatics classes and overall competency score among students. But Strahan's^26^ study at a University in USA only found a small improvement in students’ mean scores after formal informatics instruction, which was attributed to limited clinical practice exposure. These different results highlighted herein suggest that geographical context of these comparative studies in relation to informatics education and competence warrants further investigation.

## Research limitations and future directions

Our study has some limitations. Competencies were self-reported and not an assessment of students’ informatics knowledge or skills, with potential for participants to overrate their competency level.^24,30^ Non-response bias may exist due to voluntary participation of respondents. Convenience sampling and participant self-selection may not truly represent the target population. Survey recruitment was a challenge despite several invitation reminders sent to potential participants to increase participation rate and wider representation from different entry-level nursing programs. Reasons for this may be (a) increased academic workload on students rapidly transitioning to online learning and limited availability during the COVID-19 pandemic, and (b) the study invitation was online via the education platform and some potential participants may not have scrolled through the web page which typically includes multiple communications periodically. The low response rate resulted in a smaller sample size than projected and mainly from a single BN program from one institution. In future, a larger study including different entry-level programs, multiple sites and multidisciplinary research is necessary to obtain a comprehensive picture related to digital health preparedness. A cross-sectional survey of nursing schools to examine how informatics education is currently incorporated into the curricula would be beneficial to identify appropriate strategies to address gaps in current curricula. Our survey did not include open-ended questions collecting qualitative data to augment the quantitative results which need to be addressed in future research. However, this was an exploratory descriptive study using a valid and reliable informatics competency self-assessment instrument. A cross-section of undergraduates was surveyed, and findings illustrate informatics proficiency from students’ perspectives.

## Conclusion

Although increased global attention to address digital health preparation among health professionals has seen a greater focus on informatics development within undergraduate nursing curricula, our study revealed that nursing students’ perceived informatics proficiency for practice was still an issue. Given the explosion of digital technologies in health and critical need for digital capabilities among health professionals, nursing students lacked comprehensive preparedness for competent technology-integrated practice. There were gaps in vital informatics practice knowledge and skills, compounded by the lag in nurse education which had not kept pace with technology developments. These findings have implications for not just curriculum reform but also expectations around health workforce competencies on entry-to-practice.

## Supplemental Material

sj-docx-1-dhj-10.1177_20552076231179051 - Supplemental material for Baseline evaluation of nursing students’ informatics competency for digital health practice: A descriptive exploratory study Click here for additional data file. Supplemental material, sj-docx-1-dhj-10.1177_20552076231179051 for Baseline evaluation of nursing students’ informatics competency for digital health practice: A descriptive exploratory study by Kalpana Raghunathan, Lisa McKenna and Monica Peddle in DIGITAL HEALTH

sj-docx-2-dhj-10.1177_20552076231179051 - Supplemental material for Baseline evaluation of nursing students’ informatics competency for digital health practice: A descriptive exploratory study Click here for additional data file. Supplemental material, sj-docx-2-dhj-10.1177_20552076231179051 for Baseline evaluation of nursing students’ informatics competency for digital health practice: A descriptive exploratory study by Kalpana Raghunathan, Lisa McKenna and Monica Peddle in DIGITAL HEALTH

## Appendix 1

Sharma, A., Minh Duc, N. T., Luu Lam Thang, T., Nam, N. H., Ng, S. J., Abbas, K. S., . . . Karamouzian, M. (2021). A Consensus-Based Checklist for Reporting of Survey Studies (CROSS). *Journal of General Internal Medicine*, *36*(10), 3179–3187. https://doi.org/10.1007/s11606-021-06737-1

Checklist for Reporting of Survey Studies (CROSS)

**Table:**

Section/topic	Item	Item description	Reported on page #
Title and abstract			
Title and abstract	1a	State the word “survey” along with a commonly used term in title or abstract to introduce the study's design.	Abstract
	1b	Provide an informative summary in the abstract, covering background, objectives, methods, findings/results, interpretation/discussion and conclusions.	Abstract
Introduction			
Background	2	Provide a background about the rationale of study, what has been previously done and why this survey is needed.	Manuscript Page 3-4
Purpose/aim	3	Identify specific purposes, aims, goals or objectives of the study.	Manuscript Page 5
Methods			
Study design	4	Specify the study design in the methods section with a commonly used term (e.g., cross-sectional or longitudinal).	Manuscript Page 5
	5a	Describe the questionnaire (e.g., number of sections, number of questions, number and names of instruments used).	Manuscript Page 5-6
Data collection methods	5b	Describe all questionnaire instruments that were used in the survey to measure particular concepts. Report target population, reported validity and reliability information, scoring/classification procedure and reference links (if any).	Manuscript Page 5-6
	5c	Provide information on pretesting of the questionnaire, if performed (in the article or in an online supplement). Report the method of pretesting, number of times questionnaire was pre-tested, number and demographics of participants used for pretesting and the level of similarity of demographics between pre-testing participants and sample population.	Manuscript Page 6
	5d	Questionnaire if possible, should be fully provided (in the article, or as appendices or as an online supplement).	Online supplement
Sample characteristics	6a	Describe the study population (i.e., background, locations, eligibility criteria for participant inclusion in survey, exclusion criteria).	Manuscript Page 6-7
	6b	Describe the sampling techniques used (e.g., single stage or multistage sampling, simple random sampling, stratified sampling, cluster sampling, convenience sampling). Specify the locations of sample participants whenever clustered sampling was applied.	Manuscript Page 6-7
	6c	Provide information on sample size, along with details of sample size calculation.	Manuscript Page 6-7
	6d	Describe how representative the sample is of the study population (or target population if possible), particularly for population-based surveys.	Manuscript Page 6-7
Survey administration	7a	Provide information on modes of questionnaire administration, including the type and number of contacts, the location where the survey was conducted (e.g., outpatient room or by use of online tools, such as SurveyMonkey).	Manuscript Page 6-7
	7b	Provide information of survey's time frame, such as periods of recruitment, exposure and follow-up days.	Manuscript Page 7
	7c	Provide information on the entry process: →For non-web-based surveys, provide approaches to minimize human error in data entry. →For web-based surveys, provide approaches to prevent “multiple participation” of participants.	Manuscript Page 7
Study preparation	8	Describe any preparation process before conducting the survey (e.g., interviewers’ training process, advertising the survey).	Manuscript Page 7
Ethical considerations	9a	Provide information on ethical approval for the survey if obtained, including informed consent, institutional review board [IRB] approval, Helsinki declaration and good clinical practice [GCP] declaration (as appropriate).	Title page & Manuscript Page 5
	9b	Provide information about survey anonymity and confidentiality and describe what mechanisms were used to protect unauthorized access.	Manuscript Page 7
Statistical analysis	10a	Describe statistical methods and analytical approach. Report the statistical software that was used for data analysis.	Manuscript Page 7
	10b	Report any modification of variables used in the analysis, along with reference (if available).	NA
	10c	Report details about how missing data was handled. Include rate of missing items, missing data mechanism (i.e., missing completely at random [MCAR], missing at random [MAR] or missing not at random [MNAR]) and methods used to deal with missing data (e.g., multiple imputation).	Manuscript Page 7
	10d	State how non-response error was addressed.	Manuscript Page 7
	10e	For longitudinal surveys, state how loss to follow-up was addressed.	NA
	10f	Indicate whether any methods such as weighting of items or propensity scores have been used to adjust for non-representativeness of the sample.	NA
	10g	Describe any sensitivity analysis conducted.	NA
Results			
Respondent characteristics	11a	Report numbers of individuals at each stage of the study. Consider using a flow diagram, if possible.	Manuscript Page 8
	11b	Provide reasons for non-participation at each stage, if possible.	Manuscript Page 8, 16
	11c	Report response rate, present the definition of response rate or the formula used to calculate response rate.	Manuscript Page 9
	11d	Provide information to define how unique visitors are determined. Report number of unique visitors along with relevant proportions (e.g., view proportion, participation proportion, completion proportion).	Manuscript Page 8
Descriptive results	12	Provide characteristics of study participants, as well as information on potential confounders and assessed outcomes.	Manuscript Page 8, 16
Main findings	13a	Give unadjusted estimates and, if applicable, confounder-adjusted estimates along with 95% confidence intervals and p-values.	NA
	13b	For multivariable analysis, provide information on the model building process, model fit statistics and model assumptions (as appropriate).	NA
	13c	Provide details about any sensitivity analysis performed. If there are considerable amount of missing data, report sensitivity analyses comparing the results of complete cases with that of the imputed dataset (if possible).	NA
Discussion			
Limitations	14	Discuss the limitations of the study, considering sources of potential biases and imprecisions, such as non-representativeness of sample, study design, important uncontrolled confounders.	Manuscript Page 16
Interpretations	15	Give a cautious overall interpretation of results, based on potential biases and imprecisions and suggest areas for future research.	Manuscript Page 7–12
Generalizability	16	Discuss the external validity of the results.	Manuscript Page 16
Other sections			
Role of funding source	17	State whether any funding organization has had any roles in the survey's design, implementation and analysis.	Title page
Conflict of interest	18	Declare any potential conflict of interest.	Title page
Acknowledgements	19	Provide names of organizations/persons that are acknowledged along with their contribution to the research.	Title page
