# Supplementary material for: Baseline evaluation of nursing students’ informatics competency for digital health practice: A descriptive exploratory study
Source: Digit Health. 2023 May 30;9:20552076231179051. doi: 10.1177/20552076231179051 (PMC10236246; doi:10.1177/20552076231179051)
Supplement: sj-docx-1-dhj-10.1177_20552076231179051 - Supplemental material for Baseline evaluation of nursing students’ informatics competency for digital health practice: A descriptive exploratory study [file sj-docx-1-dhj-10.1177_20552076231179051.docx]

**Digital Health Student Survey**

Survey Information Page - REDCaP

Survey Title: Digital Health Readiness Student Survey

**Survey Instructions**

Digital health practice is becoming increasingly important for nurses and midwives. The purpose of this survey is to gather data about nursing students’ perceived preparedness for using digital health technologies, which includes any information and communication technologies (ICT) to improve healthcare and health outcomes. This will assist us with ensuring courses prepare students sufficiently for this aspect of their graduate roles. As a current student, your input will be key to this.

Data collected from this survey will be reported in published papers, conference presentations, study reports and doctoral thesis. Participants will not be identified, because no personal identifiable information is collected in the survey data.

The first part relates to general information. The second part involves 21 questions pertaining to nursing informatics and technology competencies relevant to work.

*Key terms*

Nursing informatics is a combination of nursing, data, and information sciences to assist management and processing of nursing data, information, and knowledge to support to nursing practice and provision of quality care.

Informatics competencies are the technical and psychomotor skills to confidently use computers, digital health devices, and specific informatics knowledge and skills to support practice.

Electronic record systems include the various information, documentation and clinical decision support systems used to assist work and the delivery of care; such as electronic health records (integrated across organisations), electronic medical records (used within an organisation or setting), personal health records (My Health Record), and electronic medication administration systems.

They survey will take approximately *10 minutes* of your time to complete.

If you no longer want to complete the questionnaire, simply close the web browser. Completing the survey implies consent. If you change your mind after clicking on the ‘Submit’ button, we cannot withdraw your responses because we cannot link who you are with your questionnaire responses.

Before you start the survey, please review the *Participant Information and Consent Statement* linked below. Research contact details are provided below if you have any questions or concerns before starting the survey.

Researcher contact details: XXXX (PhD Candidate XXXX University) Email: xxxx

Thankyou!

Questions

**General information about you**

1. Where are you currently studying?

XXXX University

XXXX University

XXXX Institute

1. Where is your nursing school located?

Metropolitan

Rural or regional

1. What is your main mode of study?

On-campus (1)

Online (2)

Blended (3)

1. What year are you in your course?

1^st^ year (1)

2^nd^ year (2)

3^rd^ year (3)

4^th^ year (double degree) (4)

1. How do you identify your sex?

Female (1)

Male (2)

Other (3)

Do not wish to disclose (4)

1. What is your age in years?

20-25 (1)

26-35 (2)

36-45 (3)

46-55 (4)

>55 (5)

**About your course**

1. To date, have you covered any informatics related content in your nursing program?

Yes (1)

No (2)

Skip to Question 9 if the answer to Question 7 is NO

1. If you have participated in informatics education in your program, was this located in: (select all that apply)

A health informatics or information management course (1)

A nursing fundamentals course (2)

Multiple courses within the program (3)

Integrated across the curriculum (4)

A seminar or lecture on informatics in a course (5)

1. Have you ever used an electronic medical record (EMR)?

Yes (1)

No (2)

Skip to Question 15 if the answer to Question 9 is NO

1. Have you used an EMR in a simulation or clinical skills laboratory?

Yes (1)

No (2)

1. Have you used an EMR as a student during clinical placement?

Yes (1)

No (2)

1. Have you used an EMR in a current or previous place of work (employment)?

Yes (1)

No (2)

1. As a student, have you been able to access an EMR system during clinical placement?

Yes (1)

No (2)

Not completed a clinical placement (3)

Show Question 14 if the answer to Question 13 is YES

1. How did you access the EMR system during clinical placement?

Via a personal student login (1)

With an RN/staff member (2)

With a clinical educator (3)

**About your own activity**

1. Have you accessed or used your own personal My Health Record?

Yes (1)

No (2)

1. Do you participate in digital online video games?

Yes (1)

No (2)

1. How frequently do you use social media or networking applications? (select all that apply)

Facebook (1)

Instagram (2)

Tiktok (3)

Twitter (4)

LinkedIn (5)

Other (6)

Responses

Very Frequently

Frequently

Occasionally

Rarely

Never

1. How frequently do you use a computer?

Several times per day (1)

Once per day (2)

Several times per week (3)

Several times per month (4)

1. How would you describe your level of computer experience?

Beginner (1)

Medium (2)

Advanced (3)

1. How did you enter your nursing program?

As a school leaver (1)

Through a graduate entry pathway (2)

As an Enrolled Nurse (3)

Other (4)

1. What is your previous highest education qualification level?

VCE or equivalent (1)

Certificate (2)

Diploma (3)

Bachelor (4)

Graduate Diploma (5)

Masters (6)

Doctorate (7)

Skip to Question 23 if the answer to Question 21 is VCE or equivalent

1. What was the discipline of your previous education qualification?

Free text……..

1. Are you also an Enrolled Nurse?

Yes (1)

No (2)

**INFORMATICS COMPETENCY SELF-ASSESSMENT**

For each competency indicator below please rate yourself at this point in your education.

Likert scale Legend: 1 = not competent; 2 = somewhat competent; 3 = competent; 4 = very competent

|  |  | 1 = not competent | 2 = somewhat competent | 3 = competent | 4 = very competent |
| --- | --- | --- | --- | --- | --- |
|  | Digital literacy (foundational information and communication technology (ICT) skills) |  |  |  |  |
|  | Uses information and communication technology (ICT) devices (e.g., computers and peripheral devices). |  |  |  |  |
|  | Uses ICT applications (e.g., emails, intranet and internet). |  |  |  |  |
|  | Performs search and critical appraisal of on-line literature and resources. |  |  |  |  |
|  | Information and Knowledge Management |  |  |  |  |
|  | Analyses, interprets, and documents pertinent nursing data and patient data using standardised nursing and other clinical terminologies (e.g., International Classification for Nursing Practice (ICNP), Nursing Intervention Classification [NIC] & Nursing Outcomes Classification [NOC]). |  |  |  |  |
|  | Assists patients and their families to access, review, and evaluate information (e.g., health websites, online support groups, etc.). |  |  |  |  |
|  | Describes the processes of data gathering, recording and retrieval, in (electronic or paper records), and identifies informational risks, gaps, and inconsistences across the healthcare system. |  |  |  |  |
|  | Articulates the significance of information standards (i.e., messaging standards necessary for interoperable electronic health records and information sharing). |  |  |  |  |
|  | Articulates the importance of standardised nursing data. |  |  |  |  |
|  | Critically evaluates data and information from a variety of sources (e.g., practice guidelines, credible relevant websites, etc.) to inform the delivery of nursing care. |  |  |  |  |
|  | Professional and Regulatory Accountability |  |  |  |  |
|  | Complies with legal and regulatory requirement, ethical standards, and organisational policies and procedures (e.g., protection of health information, privacy, and security). |  |  |  |  |
|  | Advocates for the use of current and innovative ICTs that support safe and quality care. |  |  |  |  |
|  | Identifies and reports system process and functional issues (e.g., error messages, device malfunctions, etc.). |  |  |  |  |
|  | Maintains effective nursing practice and patient safety during any period of system unavailability. |  |  |  |  |
|  | Demonstrates professional judgment must prevail in the presence of technologies designed to support clinical care. |  |  |  |  |
|  | Recognises the importance of nurses' involvement in the design, selection, implementation, and evaluation of ICT applications and systems in health care. |  |  |  |  |
|  | Use of ICT in Delivery of Patient Care |  |  |  |  |
|  | Identifies and demonstrates appropriate use of a variety of information and communication technologies (e.g., point of care systems, electronic records, etc.). |  |  |  |  |
|  | Uses decision support tools (e.g., clinical alerts and reminders) to assist clinical judgment. |  |  |  |  |
|  | Uses information and communication technologies in a manner that does not interfere with the nurse-patient relationship. |  |  |  |  |
|  | Describes the various components of health information systems (e.g., results reporting, clinical documentation, etc.). |  |  |  |  |
|  | Describes various types of electronic records used across the continuum of care (e.g., Electronic Health Record [EHR], Electronic Medical Record [EMR], Personal Health Record [PHR], Electronic Medical Administration Records [eMAR]) and their clinical and administrative uses. |  |  |  |  |
|  | Describes benefits of informatics to improve health systems and the quality of Inter-professional patient care. |  |  |  |  |
